# Establishing the Adequate Levothyroxine Dose After Total Thyroidectomy: A Systematic Review With Meta-analysis

**DOI:** 10.1210/clinem/dgaf417

**Published:** 2025-07-19

**Authors:** Isabella Chiardi, Laura Croce, Paolo Caccavale, Jacopo Bertini, Francesca Coperchini, Flavia Magri, Carlo Cappelli, Pierpaolo Trimboli, Mario Rotondi

**Affiliations:** Department of Internal Medicine and Therapeutics, University of Pavia, Pavia 27100, Italy; Department of Internal Medicine and Therapeutics, University of Pavia, Pavia 27100, Italy; Istituti Clinici Scientifici Maugeri IRCCS, Unit of Internal Medicine and Endocrinology, Laboratory for Endocrine Disruptors, Pavia 27100, Italy; Department of Internal Medicine and Therapeutics, University of Pavia, Pavia 27100, Italy; Internal Medicine, Endocrinology and Metabolic Diseases Unit, IRCCS Ospedale Galeazzi–Sant’Ambrogio, Milan 173, Italy; Department of Internal Medicine and Therapeutics, University of Pavia, Pavia 27100, Italy; Department of Internal Medicine and Therapeutics, University of Pavia, Pavia 27100, Italy; Istituti Clinici Scientifici Maugeri IRCCS, Unit of Internal Medicine and Endocrinology, Laboratory for Endocrine Disruptors, Pavia 27100, Italy; Department of Clinical and Experimental Sciences, University of Brescia, Brescia 25123, Italy; Clinic for Endocrinology and Diabetology, Lugano Regional Hospital, Ente Ospedaliero Cantonale, Lugano CH-6900, Switzerland; Department of Internal Medicine and Therapeutics, University of Pavia, Pavia 27100, Italy; Istituti Clinici Scientifici Maugeri IRCCS, Unit of Internal Medicine and Endocrinology, Laboratory for Endocrine Disruptors, Pavia 27100, Italy

**Keywords:** thyroid, levothyroxine, total thyroidectomy, hypothyroidism

## Abstract

**Background:**

Total thyroidectomy requires lifelong levothyroxine (LT4) therapy. Achieving optimal thyroid hormone replacement at the first postoperative follow-up might be harder than expected. Despite the various LT4 dose-choosing strategies tested, there is no consensus on the most effective approach to achieve early euthyroidism.

**Materials and Methods:**

We performed a systematic review and meta-analysis, including studies published between 2000 and 2024 that reported the proportion of patients achieving euthyroidism at first follow-up after total thyroidectomy. Data from 11 studies comprising 2577 patients were analyzed. LT4 dosing strategies included fixed-dose, weight-based (dose/kg), and individualized algorithm-based methods. Meta-regression and subgroup analyses were used to explore sources of heterogeneity.

**Results:**

The pooled euthyroidism rate at first follow-up was 33.9%, with high heterogeneity across studies (I^2^ = 82.68%). No dosing strategy consistently outperformed others: dose/kg methods achieved 29% euthyroidism, while fixed or algorithm-based approaches achieved 40%, though without statistical significance. Meta-regression analysis did not identify any statistically significant predictor. No significant differences emerged between patients treated for benign or malignant thyroid diseases or between retrospective and prospective study designs.

**Conclusion:**

Only about one-third of patients achieve euthyroidism at first follow-up after thyroidectomy, regardless of LT4 dosing strategy. The current guidelines recommendation of applying a pro/kg dose may not be adequate, and even personalized algorithms yield inconsistent results. Future prospective studies are needed to refine individualized dosing protocols and identify additional factors influencing LT4 requirements.

Total thyroidectomy requires lifelong replacement therapy with levothyroxine (LT4). Achieving optimal thyroid hormones replacement promptly after surgery is important but somehow challenging. Indeed, previous studies addressing this topic clearly indicate that a significant proportion of patients who are started on LT4 therapy for postthyroidectomy hypothyroidism require multiple dose adjustments over time (1-3). The clinical scenario is thus how we should choose the starting LT4 dose in patients just after total thyroidectomy to warrant the highest rate of euthyroidism at the first postsurgery evaluation. While initial investigations used fixed LT4 doses, revised and eventually adjusted at early follow-up (1, 4-6), more recent studies propose potential tailored approaches to more precisely estimate the optimal LT4 dose (3, 6-10). Current clinical guidelines (11) on the treatment of primary hypothyroidism recommend that when choosing a starting dose of LT4, several factors including patient's weight, lean body mass, etiology of hypothyroidism, degree of thyrotropin elevation, age, and comorbidities (ie, particularly cardiac diseases) should all be taken into account. Most papers suggest that in athyreotic patients, a starting LT4 dose of 1.6 to 1.8 mcg/kg of body weight (4, 12-15) would be adequate, even though other authors have advocated for a higher starting dose of 2.0 to 2.1 mcg/kg, at least in selected settings (12, 14-16).

There is general agreement that the etiology sustaining hypothyroidism impacts on the optimal LT4 dose (14, 15), making athyreotic patients, owing to the lack of residual functional thyroid tissue, more likely to require a higher LT4 dose as compared to patients with chronic autoimmune thyroiditis. Furthermore, the therapeutic target of TSH may vary in relation to the underlying condition, such as in patients with thyroid cancer who may require higher LT4 doses (ie, TSH-suppressive therapy) (17). In addition, from the patient’s standpoint, promptly achieving euthyroidism can provide clinical benefits in terms of both well-being and reduction of further clinical consultations. Although the number of studies in this field is significant, no clear and solid information is available about the optimal LT4 starting dose to be used in athyreotic subjects with no (or at least minor) need for further adjustment over time.

This study was undertaken to systematically review the literature for achieving more robust evidence on the optimal LT4 dose to be prescribed to athyreotic patients just after total thyroidectomy. The primary outcome was to assess the proportion of patients who achieve euthyroidism under the initial LT4 dose. Secondary outcomes include comparing different dose-choosing methods and stratifying results by patient setting, which may require specific TSH targets.

## Methods

### Conduct of Review

The present systematic review was conducted in accordance with the Meta-analysis of Observational Studies in Epidemiology guidelines (18). The review protocol was registered in PROSPERO (ID 1061048). The Preferred Reporting Items for Systematic Reviews and Meta-Analyses guidelines (19) were followed in reporting this study (Supplemental Material 1) (20).

### Search Strategy

A comprehensive literature search was performed using PubMed/MEDLINE and Embase databases. The search aimed to identify studies reporting the proportion of “euthyroid” patients at their first follow-up after thyroidectomy and the strategy employed to estimate the LT4 starting dose. Euthyroid patients were defined as those with TSH levels within the target range specified in each article. For benign diseases, a higher TSH range was used, whereas for malignant conditions, risk-stratified TSH targets were applied. The search was restricted to articles published in English from January 1, 2000, to October 30, 2024. The online search was conducted by the following algorithm: [(levothyroxine) or (L-thyroxine) or (LT4)] and [(thyroidectomy) or (athyreotic)] and (dose). In the attempt to expand the search, references in the retrieved articles were also screened to identify additional studies.

### Study Selection

The inclusion criteria were original studies reporting the number of patients achieving euthyroidism at the first postthyroidectomy evaluation and the criteria used to prescribe the initial LT4 dose. Exclusion criteria were studies lacking data on postthyroidectomy follow-up or underlying thyroid pathology, review articles, and outdated publications. Two independent researchers (I.C., P.C.) screened titles and abstracts based on these criteria. Subsequently, all authors independently reviewed the full text of eligible articles to confirm their inclusion in the final analysis.

### Data Extraction

For each included study, the following information was extracted independently by 2 investigators (I.C., P.C.) in a piloted form: (1) study details (authors, publication year, and country of origin); (2) patient demographics [median age, body mass index (BMI), and sex]; (3) underlying thyroid pathology (benign or malignant); (4) LT4 dosing method; (5) follow-up data for the first and second postthyroidectomy controls. Extracted data were cross-checked for accuracy, and discrepancies were resolved through discussion. When studies included 2 (or more) different populations according to the LT4 dosing strategy employed, they were reported as separate series.

### Study Quality Assessment

The risk of bias in included studies was independently assessed by 2 authors (I.C. and P.C.) according to the National Heart, Lung and Blood Institute Quality Assessment Tool for Observational Studies. Each domain was assigned a low, high, not applicable, or not reported score (21).

### Statistical Analysis

Proportion meta-analyses from at least 4 study series were performed to calculate the pooled proportion of patients with “at target TSH” among those initially enrolled with the same LT4 starting dose strategy. The proportion calculation was repeated considering specific subgroups. Heterogeneity was assessed by using I^2^, and a value ≥50% defined the presence of heterogeneity. A random-effects model was used. Pooled data were reported with 95% confidence intervals. When heterogeneity was found, it was explored by subgroup and meta-regression analyses using several covariates. In subgroup analyses, a significant difference was defined when the 95% confidence interval of the 2 groups did not overlap. Statistical significance was set at *P* < .05. Statistical analyses were performed using Open Meta [Analyst] software (open-source software developed by the Center for Evidence Synthesis in Health, Brown University).

## Results

### Eligible Articles

**Figure 1.**
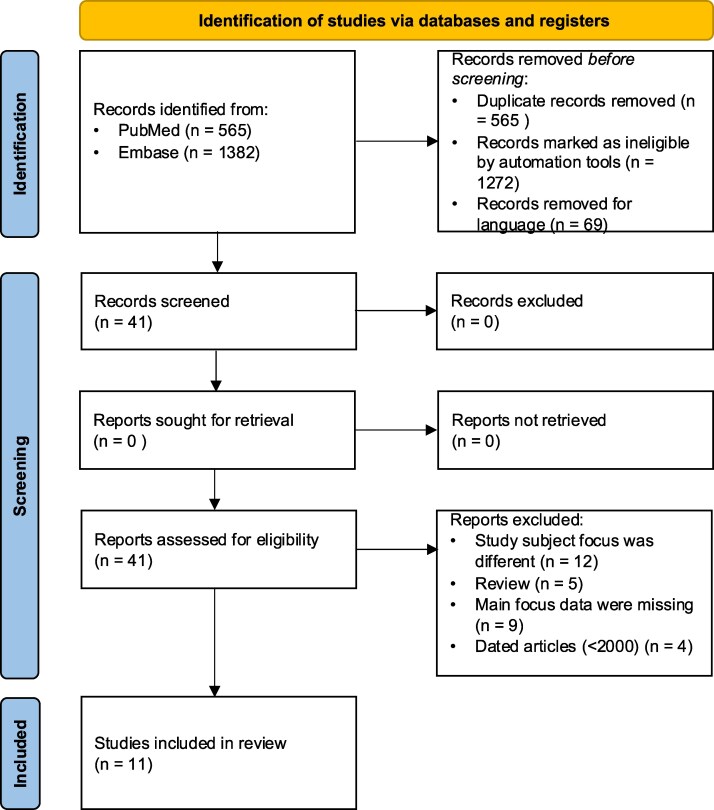
Preferred Reporting Items for Systematic Reviews and Meta-Analyses 2020 flow diagram of article search.

A total of 1947 articles were identified through the search strategy. After applying the selection criteria, 34 studies were found to be relevant to the study focus, and ultimately 11 articles were included in the final review (1, 3-6, 8-10, 22-24). Figure 1 illustrates the search flow.

Table 1 summarizes the main study details.

**Table 1.** General characteristics of the studies included in the present systematic review

Reference	Authors	Year	Country	No. of patients	Median age (yrs)	Sex F/M	Median BMI	Thyroid pathology	LT4 starting dose used
	Fixed dose*^a^*								
4	Olubowale and Chadwick	2005	UK	98	46	86/12		Benign	100 mcg
1	Mistry et al	2011	UK	100	43	78/22	27	Benign	100 mcg
5	Verhaert et al	2006	Belgium	48				Benign	150 mcg
	Dose/kg*^b^*								
3	Di Donna et al (population 1)	2014	Italy	92	51.3	72/20	25.2	Benign	1.6
22	Ojomo et al	2013	USA	122	49	101/21	30.5	Benign	1.6
8	Elfenbein et al (population 1)	2016	USA	150	50	126/24	29.9	Benign	1.6
23	Barrio et al (population 1)	2023	USA	289				Benign	1.6
23	Barrio et al (population 2)	2023	USA	414				Malignant	1.6
23	Barrio et al (population 3)	2023	USA	113				Malignant	1.8
23	Barrio et al (population 4)	2023	USA	135				Malignant	2
24	Zhou et al	2023	China	551	43.93	425/126	23.7	Malignant	1.6
10	Lu et al (population 1)	2019	Philippines	26	47.6	23/3	25.5	Malignant	2.2
	Algorithm-based*^c^*								
3	Di Donna et al (population 2)	2014	Italy	31	47.4	26/5	24.44	Benign	Nomogram based on age and BMI
8	Elfenbein et al (population 2)	2016	USA	180	48	153/27	30.4	Benign	BMI based protocol
9	Papoian et al	2019	USA	114	55.8	96/18	31.1	Benign	Regression analysis
10	Lu et al (population 2)	2019	Philippines	26	45.4	26/0	27.6	Malignant	Protocol based on LBW
6	Liu et al	2024	China	88	40.22	63/25	26.52	Malignant	SVR model

Abbreviations: BMI, body mass index; LBW, lean body weight; SVR, support vector regression (based on body surface area, weight, hemoglobin, height, BMI, and age).

^*a*^Standard dose for all patients.

^*b*^Dose calculated according to a standard multiplier (typically 1.6 × body weight in kg for benign disease, and ranging from 1.6 to 2.2 for malignant thyroid pathology).

^*c*^Complex dosing strategies that incorporate additional variables beyond body weight, such as age, BMI, and clinical parameters. Complete details on algorithms are reported in Supplemental Material 2.

### Assessment of Study Quality

Supplemental Material 2 (20) illustrates the risk of bias in the included studies. Overall, 11 out of the 11 observational studies were assessed as having a low risk of bias across all evaluated criteria. Considering the study's aim and design, item 12 regarding the outcome assessors being blinded to the exposure status of participants was deemed not applicable. No studies provided information regarding power or sample size justification.

### Qualitative Analysis (Systematic Review)

This systematic review includes 11 papers published between 2005 and 2024 (1, 3-6, 8-10, 22-24). Six studies were observational with retrospective data analysis (4, 9, 10, 22-24), 2 had a prospective data analysis (1, 5), and 3 studies did both a retrospective and a prospective analysis (3, 6, 8). Four studies were published by European institutions [2 from UK (1, 4), 1 from Italy (3), and 1 from Belgium (5)], 3 were from Asia [2 from China (6, 24) and 1 from the Philippines (10)], and 4 from North America [US (8, 9, 22, 23)]. The study involved 2577 patients who underwent thyroidectomy for different reasons: 7 studies included only benign pathologies (1, 3-5, 8, 9, 22), and 3 studies included only malignant diseases (6, 10, 24); 1 study analyzed both categories (23). The mean age of patients (from studies reporting such information) ranged from 40 to 56 years and the mean BMI from 23.7 to 31.1 kg/m^2^, with an overall female-to-male ratio of about 4.38:1. While all studies reported mean age of included patients, only 2 studies (1, 3) stratified results according to age, although using different age thresholds. Mistry et al (1) stratified patients according to age quartiles (<32, 32.1-42, 42.1-53.5, >53.5). Di Donna et al (3) assigned patients to 3 groups (<40, 40-55, >55 years). Some but not all studies considered clinical factors potentially impacting LT4 requirements. In particular, 5 studies (6, 9, 10, 22, 23) reported pregnancy or lactation during follow-up as an exclusion criterion. Two studies excluded patients treated with medications associated with thyroid hormone malabsorption or metabolism (3, 10), while another study included them but analyzed them separately (23). Two studies excluded patients with a history of gastric diseases potentially causing malabsorption (3, 10), while another study excluded only patients who had undergone a gastric bypass (22). Only 2 studies reported major cardiovascular, hepatic, or renal diseases as possible confounding factors for LT4 dose calculation (10, 24). No study evaluated genetic polymorphisms affecting thyroid hormone transporters or deiodinases.

All studies were conducted in referral hospital centers. The normal TSH range was reported by all studies, with most using 0.4 to 4.5, while only 1 study applied a 0.4 to 2.5 range (3). For tumors, different risk categories were considered: low risk (0.5-2), intermediate risk (0.1-0.5), and high risk (<0.1). Of the 11 studies included, 7 analyzed patients using a single LT4 dosing method: 2 studies used a dose/kg approach (22, 24), 3 applied a fixed-dose regimen (1, 4, 5), and 2 employed an algorithm-based method (6, 9). In the remaining 4 studies, multiple groups of patients received different dosing strategies: 3 (3, 8, 10) compared 2 groups (1 using a dose/kg method and the other an algorithm-based approach) and 1 (23) examined 4 groups using a dose/kg method but with a different TSH target according to their disease.

As a result, the 11 studies yielded 17 patient groups. Among them, 3 groups followed a fixed-dose regimen [2 receiving 100 mcg (1, 4), 1 receiving 150 mcg (5)], 9 groups used a dose/kg approach (3, 8, 10, 22-24), and 5 groups applied an algorithm-based calculation (3, 6, 8-10).

A detailed description of the algorithm-based approaches is reported in Supplemental Material 3 (20).

All studies reported the number of euthyroid patients at the first control, which was typically conducted at 6 to 8 weeks, with the widest timeframe being 4 to 15 weeks.

### Quantitative Analysis (Meta-analysis)

**Figure 2.**
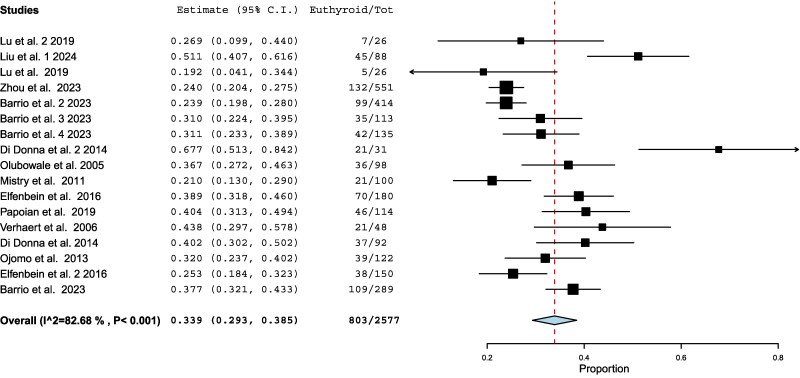
Forest plot meta-analysis of different studies assessing dosing strategies. Each square represents the weight of the study, while the diamond represents the pooled result, with its width indicating the 95% confidence interval.

The pooled proportion of patients achieving target thyroid hormone levels at the first postthyroidectomy follow-up was 33.9% (803/2577 patients), with a confidence interval from 29.3% to 38.5% (Fig. 2). The analysis included 17 different study populations, demonstrating substantial heterogeneity (I^2^ = 82.68%). A comparative analysis based on key study characteristics was performed to explore potential sources of heterogeneity (Table 2).

**Table 2.** Pooled results of different dosing strategies for achieving euthyroidism, including the number of studied groups and patients, euthyroid rates with 95% CI, and heterogeneity

Strategy of dosing method	No. of groups studied	No. of patients	Euthyroid (%)	95% CI	Heterogeneity (I^2^)
All groups	17	2577	33.9	29.3-38.5	82.68
Dose/kg	9	1892	29.4	25.1-33.7	73.16
Fixed + algorithm	8	685	40.2	31.5-48.9	81.91
Benign thyroid pathology	10	1224	36.9	31-42.7	78.06
Malignant thyroid pathology	7	1353	29.5	23.4-35.5	78.67
Prospective studies	5	447	43.5	29.6-57.4	82.68
Retrospective studies	12	2130	30.7	26.7-34.8	72.45
Dose/kg + BMI + LBM based methods	12	2212	31.1	26.9-35.2	75.53
Fixed dose or complex algorithm (not only BMI/LBM based)	5	365	43.2	29.3-58.1	89.11

Abbreviations: BMI, body mass index; CI, confidence interval; LBM, lean body mass.

**Figure 3.**
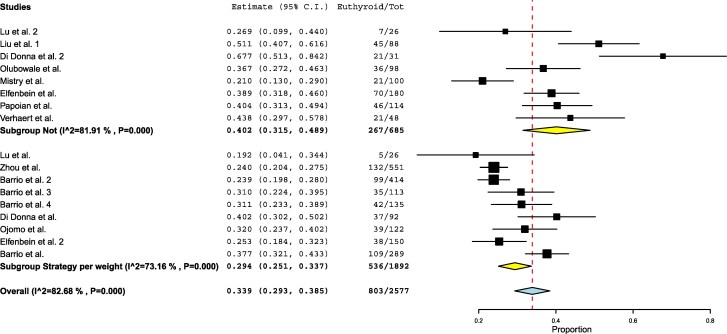
Forest plot meta-analysis comparing the dose/kg approach vs fixed-dose or algorithm-based approaches. Each square represents the weight of the study, while the diamond represents the pooled result, with its width indicating the 95% confidence interval.

When comparing studies using a dose/kg approach vs those applying a fixed-dose or algorithm-based approach, a slight difference in median achievement rates was observed, with 29.4% in the dose/kg group compared to 40.2% in all other studies with no significant difference (Fig. 3). However, the dose/kg group exhibited lower variability, as indicated by a narrower confidence interval, whereas heterogeneity was higher in the fixed-dose/algorithm-based group (I^2^ = 73% vs 81%).

**Figure 4.**
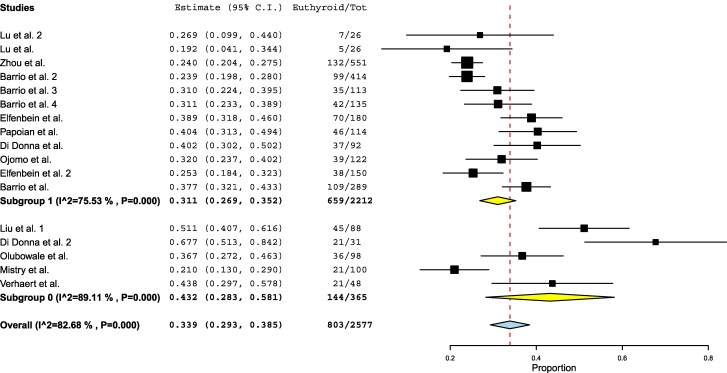
Forest plot meta-analysis comparing studies using a dose/kg approach or incorporating body mass index, lean body mass, or age vs all other approaches (fixed dose or complex algorithms). Each square represents the weight of the study, while the diamond represents the pooled result, with its width indicating the 95% confidence interval.

A further comparison was performed between studies applying a dose/kg approach or any approach that incorporated BMI, lean body mass, or age vs those that did not (Fig. 4). Although achievement rates were slightly higher in the studies that did not account for these variables (31% vs 43%), the difference remained nonsignificant. In fact, heterogeneity was substantially higher in this second group (I^2^ = 75% vs 89%).

**Figure 5.**
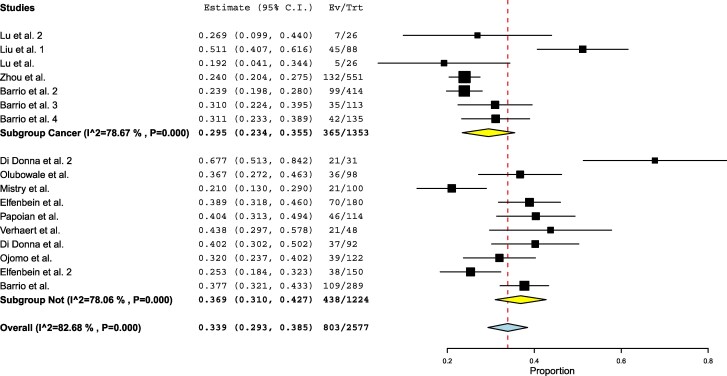
Forest plot meta-analysis comparing the benign thyroid pathology group vs the malignant thyroid pathology group. Each square represents the weight of the study, while the diamond represents the pooled result, with its width indicating the 95% confidence interval.

No significant differences were found when comparing studies with small vs large sample sizes. Similarly, no significant differences were observed when analyzing retrospective vs prospective studies. The comparison between patients treated for benign vs malignant thyroid diseases also revealed no significant difference in target TSH achievement rates (Fig. 5).

### Meta-regression Analysis

Meta-regression analyses using BMI, age, sample size, and various dose/kg regimens as covariates—with the rate of target achievement as the dependent variable—did not yield any statistically significant results (see Supplemental Material 4) (20).

## Discussion

As millions of people around the world take LT4 daily and a huge number of patients with goiter or cancer undergo thyroidectomy, achieving robust data about the optimal starting LT4 dose should be important for helping the clinical practice of thyroidologists. The main goal of the present meta-analysis was to assess whether different approaches for a LT4 dose-choosing strategy would translate into a higher number of euthyroid patients at the first follow-up visit after total thyroidectomy.

The results of the present analysis show a mean percentage of euthyroid patients receiving LT4 at the first follow-up after thyroidectomy of ∼34%. This indicates that, in most patients, independently from the LT4 dose-choosing method used, TSH level was either below or above the normal range. The high heterogeneity in terms of euthyroidism rates observed across studies (ranging from 19% to 68%) using different dose-choosing strategies highlights the complexity of choosing an adequate starting LT4 replacement dose in thyroidectomized patients. We aimed to identify the primary contributors to this variability, considering methodological differences in LT4 dosing and patient characteristics by assessing whether different methods for estimating LT4 dose would lead to different results in terms of rates of euthyroidism achieved.

The studies included in our analysis employed different dosing approaches, including fixed-dose regimens, weight-based dosing (dose/kg), and individualized algorithms incorporating factors such as BMI, lean body mass (LBM), or age. We conducted mainly 2 comparative analyses: (1) dose/kg studies vs all other approaches and (2) weight-based, BMI-based, LBM-based dosing vs fixed-dose and other particular algorithm-based methods. In both analyses, the second group exhibited higher rates of euthyroidism; however, this was accompanied by greater heterogeneity and wider confidence intervals, primarily due to 1 study belonging to the second group reporting a 68% achievement rate among 31 patients. When considering how these factors were incorporated into dose-choosing algorithms, we found that only 1 study included LBM in its algorithm, yet it achieved a euthyroidism rate of just 26%, indicating no clear advantage over other methods. No study accounted for sex in the dose-choosing algorithm. Two studies (3, 6) included age as a variable, reporting euthyroidism rates of 67% and 58%. Three studies (3, 6, 8) incorporated BMI into their algorithms. Two studies (3, 6) considered age and BMI, while 1 used a BMI-based approach (8), achieving a euthyroidism rate of 38%.

It is also important to note that there were studies we had to exclude from the analysis, such as those by Zaborek et al (2) and Valenzuela et al (7), which proposed algorithms based on a retrospective analysis of their findings and hypothesized that these algorithms could bear favorable results. However, they examined a putative population without subsequent real-world testing.

Currently available literature data suggests that several patient-specific factors (ie, body weight, BMI, LBM, age, and sex) impact LT4 dose needs (11, 13, 25). However, the absence of a statistically significant association between BMI or age and target achievement in the meta-regression [Supplementary Material 4 (20)] highlights that BMI-based and age-based LT4 dosing alone is insufficient to warrant higher rates of euthyroidism, reinforcing the idea that multiple factors contribute to dosing variability. Furthermore, the present analysis included populations treated for both benign and malignant thyroid conditions. Although one might assume that different TSH targets could influence achievement rates, our analysis found no significant differences between patients treated for benign or malignant thyroid diseases, suggesting that even with lower TSH targets, achieving optimal levels remains challenging.

By trying to translate into real-world clinical practice the results of the present meta-analysis, the following points appear of potential relevance:

- The rate of patients achieving euthyroidism at the first postsurgery evaluation is rather low (mean 33.9%), independently from the specific LT4 dose-choosing strategy.
- Great heterogeneity was found across the included studies, in terms of study design, study population, and therapeutic intervention. However, rather than a limitation, this should be regarded as a strength for the meta-analytic approach, since it underscores the need for more standardized and well-designed studies. Furthermore, this approach allowed us to not only synthesize the current body of evidence but also highlight the great heterogeneities among studies, supporting the final message that there are currently no LT4 dose-choosing strategies that perform better than others.
- The included studies lacked, in most cases, detailed information as to potential confounders including comorbidities, concomitant medications, patient's compliance, malabsorption, and genetic polymorphisms affecting thyroid hormone transporters or deiodinases. This might represent a limitation, but it should be highlighted that, due to the following considerations, this has not played a major confounder role. Indeed, included studies took into account general populations of thyroidectomized patients, which would translate into the fact that potentially “confounding conditions” would be equally represented in all the case series included and likely equally represented in studies adopting different strategies. Furthermore, it could be reasonably assumed that the vast majority of patients included in all of the studies would actually be free of potential confounders. Thus we could conclude that these confounding factors would not impact the overall final outcome.

Taking together the reported findings, the current guideline recommendation of a standard 1.6 µg/kg LT4 dose (11) does not appear to be fully supported by evidence.

According to the study aim, the results of the present systematic review show that just above one-third of athyreotic patients have normal TSH at their first postsurgery follow-up independently of the strategy used to decide the LT4 starting dose. Despite LT4 therapy being a well-established and safe treatment, achieving euthyroidism at the first postthyroidectomy follow-up remains challenging. Although some differences in terms of euthyroidism rates were reached, previously tested dosing strategies ranging from very simple ones (1, 4, 5) to rather complex algorithms (3, 6, 8-10) do not fully account for interindividual variability, underscoring the limitations of a one-size-fits-all approach. Further investigation is needed to uncover these factors and develop more precise, individualized dosing algorithms to improve postthyroidectomy management. Future well-controlled and adequately sized prospective studies specifically designed to address this issue should take into account specific participant eligibility criteria including age, BMI, body composition, underlying thyroid disorders, concomitant therapies, causes of malabsorption, comorbidities, and patient compliance.

## Data Availability

All datasets generated during and/or analyzed during the current study are not publicly available but are available from the corresponding author on reasonable request.
